# Factors affecting urinary continence and sexual potency recovery after robotic-assisted radical prostatectomy

**DOI:** 10.1590/S1677-5538.IBJU.2018.0704

**Published:** 2019-09-02

**Authors:** Mark Fernando Neumaier, Carlos Henrique Segall, Marcelo Hisano, Flávio Eduardo Trigo Rocha, Sami Arap, Marco A. Arap

**Affiliations:** 1 Hospital Sírio-Libanês, São Paulo, SP. Brasil;; 2 Hospital das Clínicas da Faculdade de Medicina da Universidade de São Paulo, São Paulo, SP, Brasil

**Keywords:** Prostatic Neoplasms, Adenocarcinoma, Urinary Incontinence

## Abstract

**Introduction:**

Robot-assisted radical prostatectomy (RARP) is the most recent surgical technique for localized prostate cancer. The Da Vinci (Intuitive Surgical, Sunnyvale, CA) system was first introduced in Brazil in 2008, with a fast growing number of surgeries performed each year.

**Objective:**

Our primary endpoint is to analyze possible predictors of functional outcomes, related to patient and tumor features. As secondary endpoint, describe functional outcomes (urinary continence and sexual potency) from RARP performed in the Sírio-Libanês Hospital (SLH), a private institution, in São Paulo, from April 2008 to December 2015.

**Materials and Method:**

Data from 104 consecutive patients operated by two surgeons from the SLH (MA and SA) between 2008 and 2015, with a minimum 12 months follow-up, were collected. Patient features (age, body mass index - BMI, PSA, date of surgery and sexual function), tumor features (tumor stage, Gleason and surgical margins) and follow-up data (time to reach urinary continence and sexual potency) were the variables collected at 1, 3, 6 and 12 month and every 6 months thereafter. Continence was defined as the use of *no pad* on medical interview and sexual potency defined as the capability for vaginal penetration with or without fosphodiesterase type 5 inhibitors.

**Results:**

Mean age was 60 years old and mean BMI was 28.45 kg/m2. BMI >30kg/m2 (p<0.001) and age (p=0.011) were significant predictors for worse sexual potency after surgery. After 1, 3, 6 and 12 months, 20.7%, 45.7%, 60.9% and 71.8% from patients were potent, respectively. The urinary continence was reached in 36.5%, 80.3%, 88.6% and 92.8% after 1, 3, 6 and 12 months, respectively. Until the end of the study, only one patient was incontinent and 20.7% were impotent.

**Conclusion:**

Age was a predictor of urinary and erectile function recovery in 12 months. BMI was significant factor for potency recovery. We obtained in a private hospital good functional results after 12 months of follow-up.

## INTRODUCTION

Prostate cancer is the most common solid organ neoplasm in men and the second cause of cancer death in Brazil. Estimates of the National Cancer Institute (INCA) for the year 2016 predict 61,200 new cases. In 2013, 13,770 deaths by prostate cancer were confirmed (1).

Since Walsh’s nerve sparing technique, radical prostatectomy has become the preferred method for prostate cancer treatment (2). Robot-assisted laparoscopic radical prostatectomy (RARP) was introduced in 2001 and quickly spread in USA. In 2008, the first RARP was performed in Brazil. The benefit of RARP on functional outcomes was demonstrated first by Tewari et al., who reported a faster potency and continence recovery over open radical prostatectomy (3).

Quality of life after surgery is strictly related to continence and potency recovery (4). Therefore, besides cancer control, functional outcomes have been studied in an attempt to understand which patients may be at increased risk. Pre-operative orientation could improve patient satisfaction and quality-of-life. Furthermore, the risk of any sequel discourages many patients and surgeons considering radical prostatectomy.

To our knowledge, there is no national publication reporting predictors of functional outcomes recovery after RARP with more than one hundred patients. The main objective of this study is to analyze predictors of functional outcomes recovery after robot-assisted radical prostatectomy in a private hospital in Brazil.

## MATERIALS AND METHODS

After approval by the Research Ethics Committee of the Sírio Libanês Hospital, medical and hospital records data were collected from patients submitted to robot-assisted laparoscopic prostatectomy between April 2008 and December 2015 by two surgeons of the Sírio Libanês Hospital (SA. e MA), a private hospital in São Paulo, Brazil.

All patients have been diagnosed with adenocarcinoma of the prostate by biopsy of the prostate guided by transrectal ultrasonography with at least 12 fragments. Only patients with clinically localized disease, life expectancy greater than 10 years and postoperative follow-up of at least 12 months, were included. Patients with previous history of radiotherapy or any neoadjuvant therapy were excluded.

The data collected were age, body mass index (BMI), sexual function prior to the procedure (by interview), PSA (ng/dL, at the time of diagnosis), prostate size – weighing of the piece performed by the pathologist (grams), date of surgery, comorbidities (hypertension, dyslipidemia, diabetes and smoking; based on the use of medications and previous medical history), Gleason of the surgical piece, pathological stage (2002 TNM of the *American Joint Committee on Cancer/Union for International Cancer Control*), (5) sexual potency recovery time (weeks; defined as the capacity for vaginal penetration, with or without the use of phosphodiesterase-5 inhibitors) and urinary continence (weeks; defined as the full continence capacity, without the use of absorbents).

Surgeries were performed according to the technique described by Patel (6). Most of the time, Rocco’s point and bilateral nerve sparing (BNS) were performed when possible. Patients with a suspicious capsule invasion in either side of the prostate were usually not submitted to the BNS, in order to prevent positive surgical margin. The patient was discharged 2 to 3 days after the procedure and the bladder catheter was removed on the 10th postoperative day. Patients were instructed by the surgeons on sphincter rehabilitation the day the catheter was removed and we stimulated the use of iPD-5 for all patients after the first post-operative month, either with tadalafil 5mg on a daily basis, or sildenafil 50mg three times a week.

### Statistical analysis

Continuous quantitative variables were described by measures of central tendency and dispersion, while categorical variables were described by means of absolute and relative frequencies (percentages).

The time to reach potency and continence was assessed through Kaplan-Meier. The comparison of the curves was done by the log-rank test for categorical variables. Patients who did not reach potency / continence according to the pre-established criteria (see methods), until the last consultation, were considered impotent and incontinent.

Cox regression models were constructed to identify independent predictors of urinary continence and sexual potency for continuous variables. Only the variables that reached p<0.25 in the analysis were included in the multivariate analysis.

The results of the statistical tests were considered significant when p<0.05. All variables were entered into a database and analyzed using the R statistical program (R Core Team, 2014).

## RESULTS

Between 2008 and 2016, 104 consecutive robot-assisted laparoscopic radical prostatectomies were performed at the Sírio Libanês Hospital by surgeons SA and MA. The average age was 60 years (35-80 years) and the average BMI was of 28.45 kg/m^2^ (±4.2 kg/m^2^). The remaining descriptive (Table-1) and categorical (Table-2) characteristics of the series of patients are described below.

**Table 1:** Descriptive summary of continuous characteristics.

	N	Min	Median	Max	Mean	SD
Age	104	35	61	80	60.05	8.34
BMI	62	19.08	28.39	42.93	28.45	4.2
PSA	103	0.88	5.6	41	6.48	4.67

**N** = Sample size; **SD** = Standard Deviation

**Table 2:** Descriptive summary of categorical characteristics.

	Quantity	Percentage
**BMI (Kg/m ^2^)**		
<30	46	74.2
>30	16	25.8
**PSA (mg/dL)**		
<10	90	87.4
10-20	11	10.7
>20	2	1.9
**Gleason Total**		
6	17	16.3
7	62	59.6
8-9	25	24
**Margin**		
Negative	89	85.6
Positive	15	14.4
**Extraprostatic Extension**		
No Impairment	78	75
Focal Impairment	22	21.2
Extensive Impairment	4	3.8
**Seminal Vesicles Impairment**		
Negative	97	94.2
Positive	6	5.8
**Preservation of Bundles**		
No Preservation	4	4.8
With Unilateral Preservation	24	28.6
With Bilateral Preservation	56	66.7
**Pathological Stage**		
pT2	78	75
pT3	26	25

Data from 96 and 94 patients were analyzed for continence and potency, respectively. Two patients did not return to all the scheduled appointments and 6 patients had important data missing from their charts. Another 2 patients were excluded from potency analysis as they started androgen deprivation therapy (ADT) before 6 months of follow-up.

None of the analyzed continuous variables (BMI, PSA, D’Amico risk, presence of positive margins, prostate volume, BNS, age and comorbidities) were shown to be related to continence recovery in the univariate analysis (Table-3). However, when age was analyzed as a categorical variable (>60 years old), it was significant (p=0.03).

**Table 3:** – Descriptive data according to continence and potency status.

	Incontinence	Impotence
	Quantity/Total (%)	Quantity/Total (%)
	
**BMI (Kg/m^2^)**	**p=0.294**	**p<0.001**
<30	3/45 (6.7)	35/43 (81.4)
>30	3/15 (20)	6/15 (40)
**PSA (mg/dL)**	**p=0.91**	**p=0.229**
<10	7/85 (8.2)	60/84 (71.4)
10-20	2/11 (18.2)	6/9 (66.7)
>20	0/2 (0)	0/2 (0)
**Gleason Sum**	**p=0.355**	**p=0.976**
6	1/16 (6.2)	12/15 (80)
7	5/60 (8.3)	42/60 (70)
8-9	3/23 (13)	13/21 (61.9)
**Margins**	**p=0.431**	**p=0.48**
Negative	7/85 (8.2)	62/82 (75.6)
Positive	2/14 (14.3)	5/14 (35.7)
**Extra-prostatic extension**	**p=0.454**	**p=0.676**
No extension	6/76 (7.9)	54/73 (74)
Focal extension	2/20 (10)	13/21 (61.9)
Larger extension	1/3 (33.3)	0/2 (0)
**Seminal Vesicles**	**p=0.404**	**p=0.815**
Negative	8/92 (8.7)	63/90 (70)
Positive	1/6 (16.7)	3/5 (60)
**Neurovascular Bundles**	**p=0.678**	**p=0.145**
No preservation	0/4 (0)	0/3 (0)
Unilateral preservation	3/20 (15)	11/20 (55)
Bilateral preservation	6/55 (10.9)	41/54 (75.9)
**Pathologic stage**	**p=0.8**	**p=0.75**
pT2	6/76 (7.9)	54/73 (74)
pT3	3/23 (13)	13/23 (56.5)

**Figure 1:**
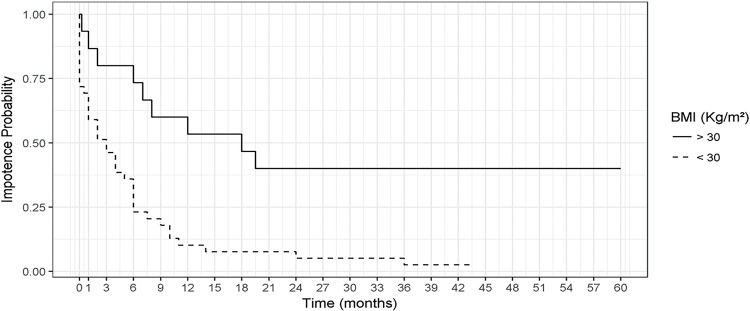
– Estimates of the probability of impotence considering BMI - Kaplan Meier Estimates.

In the univariate analysis, BMI (p<0.001) and age (p=0.11) were statistically significant factors that influenced potency recovery. They also remained statistically significant in the multivariate analysis. Figure-1 illustrates the likelihood of remaining impotent according to BMI over time. After 24 months, no patient with BMI >30 kg/m^2^ regained sexual potency.

Hypertension, diabetes, smoking, dyslipidemia and the presence of comorbidities also did not influence the recovery of sexual potency. The evaluation of age as a categorical variable wasn’t statistically significant.

**Figure 2:**
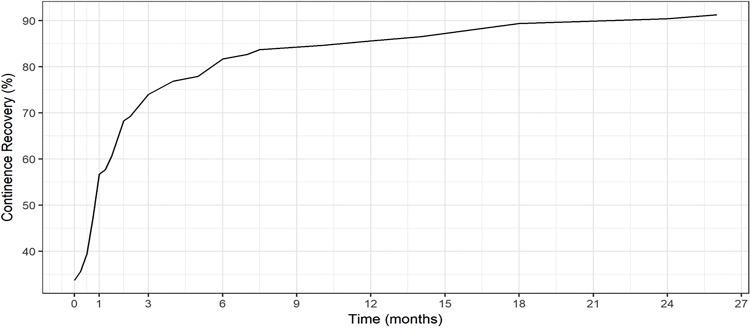
– Relation of percentage of continent patients with time (months).

The frequency of continent patients immediately after removal of the bladder catheter and after 3, 6 and 12 months were of 36.5%; 80.3%; 88.6% and 92.8%, respectively. After the first year, 6 patients who were incontinent (6.2%) recovered urinary continence by the end of the evaluated period (Table-4). Figure-2 shows the evolution of urinary continence recovery over time. The average time for continence recovery was 2.66 months.

**Table 4:** – Recovery of urinary continence and sexual potency.

	Number	Absolute percentage (%)	Acumulated percentage (%)
**Time to urinary continence recovery**			
Immediate	35	36.5	36.5
3 months	42	43.8	80.3
6 months	8	8.3	88.6
12 months	4	4.2	92.8
Over 12 months	6	6.2	99
**Time to sexual potency recovery**			
Immediate	19	20.7	20.7
3 months	23	25	45.7
6 months	14	15.2	60.9
12 months	10	10.9	71.8
Over 12 months	7	7.6	79.4
No recovery	19	20.7	100

**Figure 3:**
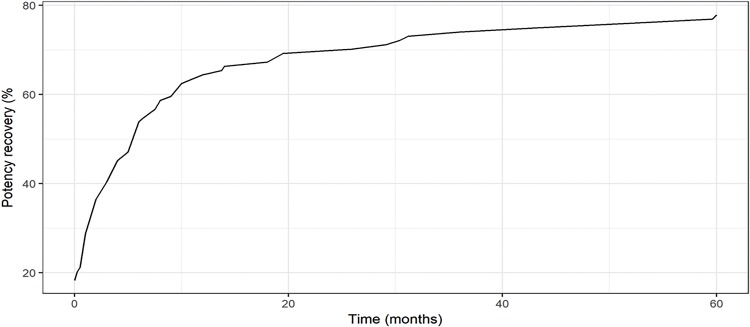
– Percentage of potent patients over time (months).

Recovery of sexual potency occurred as early as the first month for 20.7% of patients. In the course of 3, 6 and 12 months, 45.7%; 60.9% and 71.8% recovered potency, respectively (Table-4 and Figure-3). The average time to recover sexual potency was 7.72 months. Nineteen patients (20.7%) remained impotent.

## DISCUSSION

Urinary incontinence is considered the complication that most affects the patient’s quality of life (7). After the first year of follow-up, more than 90% of the patients were continent (*no pad*) in this series. The average time for continence recovery was 2.66 months. Our continence results, at the end of the first year after surgery, are within the range observed in the systematic review and meta-analysis of Ficarra et al*.,* which showed an average incidence of urinary incontinence at 12 months of 9% (8%-11%), despite considering continent patients using up to 1 pad per day (8). Another multi-institutional study with 1,812 patients showed incontinence rates of 21% (considering 0 pad) after 12 months (9). The number of patients who reached urinary continence immediately after the withdrawal of the bladder catheter (36.5%) was also in agreement with the literature, which varies from 13.1% - 68.9% (3, 10, 11).

We chose to include in the study patients with a minimum of 12 months of follow-up, since it is the period in which most patients recover sexual function and urinary continence. Some patients in the present study regained urinary continence after more than 2 years of follow-up. In a series by Ficarra et al.*,* there was no continence recovery after 12 months (12), although another study showed that a slow recovery may be the case (13).

Among the possible predictors of urinary continence that have been reported (age, obesity, length of the membranous urethra, anastomotic stricture, experience of the surgeon, neurovascular bundle preservation, large prostate volume, obstructive urinary symptoms and the preservation of the bladder neck) (14), age is one of the most consistent (15). Most published series have shown that young patients (<60 years old) present faster recovery and better results in 12 months (16-19). Lavigueur-Blouin et al. evaluated the predictive factors for early recovery (up to 1 month) of continence after RARP, where 57% of patients younger than 55 years of age were continent in the first month and only 33% of patients with more advanced age (20). In our series, age as a categorical variable (≤ 60 years old and >60 years old) was also a predictor of continence.

Obesity (BMI ≥ 30 kg/m^2^) has also been reported as an adverse prognostic factor in radical prostatectomy. In a recent review study, BMI was responsible for longer surgical time, greater surgical bleeding and worse functional results (21). Wiltz et al. published one of the largest series, with 945 patients stratified using BMI into normal (<25 kg/m^2^), overweight (≥25 and <30 kg/m^2^) and obese (≥30 kg/m^2^) (13). Patients with normal BMI presented better continence results compared to more obese patients after 12 months (70% vs. 68% vs. 57%, p=0.03) and 24 months (75% vs. 71% vs. 57%, p=0.04). Ahlering et al. also reported worse results for obese patients, being 47% of patients with BMI≥30 kg/m^2^ and 91.4% of patients with BMI<30 kg/m^2^ continent (0 pad) after 6 months (p≤0.001) (22). In our series, 16 patients presented BMI ≥30 kg/m^2^ at the time of surgery (15 with complete data), and there was no statistical difference in recovery of urinary continence compared to patients with BMI <30 kg/m^2^. Although it did not reach statistical significance, we observed that the average time to reach urinary continence was almost double for obese patients (5.08 months vs. 2.71 months). This may be explained due to the small number of obese patients in the series.

After 1, 3, 6 and 12 months, there were 20.7%, 45.7%, 60.9% and 71.8% of patients who recovered sexual potency, respectively. After 12 months, another 7 patients regained sexual potency and in

In 19 cases (20.7%) there was no recovery of sexual function. The average time to recover sexual potency was 7.72 months. Shikanov et al. reported similar results using the interview made by the surgeon. After 3, 6 and 12 months, results were 57%, 63% and 82%, respectively (23). Using SHIM (*Sexual Health Index for Men*) questionnaire, a Canadian study with 722 cases reported recovery of sexual potency in 1, 3, 6 and 12 months of 19.5%; 31.4%; 37.2% and 52.4% (24).

The relationship between BMI and sexual potency recovery after RARP is suggested in some studies, but it is still controversial (25, 26). It is intuitive that the presence of a greater amount of periprostatic adipose tissue may increase the chance of injury to the neurovascular bundle. Obesity is also associated with the metabolic syndrome, the use of medications that may affect the quality of the erection, in addition to an endothelial inflammation and dysfunction (27). In the series of Wiltz et al. the results were significantly worse after RARP for obese patients when compared to overweight (BMI≥25kgm^2^ and <30kgm^2^) and normal patients (BMI<25kgm^2^), with 48.4%, 59.6% and 68.5% of potent patients after 12 months, and 55.9%, 78.9% and 80.3% after 24 months, respectively (p=0.02) (13). Wiltz et al. analysis considered only bilateral bundle preservation surgeries (13). The association between BMI ≥30 kg/m^2^ and erectile dysfunction 12 months after RARP (p<0.001) found in our study may be related to the prior committed sexual function and the presence of other comorbidities that were not evaluated.

The association between comorbidities that increase cardiovascular risk such as diabetes, hypertension, dyslipidemia, and smoking with erectile dysfunction is well recognized (28). Such diseases could also influence the recovery of sexual potency after RARP (29). Isolated comorbidities or the presence of any of them (hypertension, diabetes, dyslipidemia and smoking history) were not significant for the recovery of both potency and urinary continence. It is important to emphasize that we considered smokers all patients with previous or current or smoking history and we did not quantify packs consumed per year and time.

Our study presents a number of limitations inherent to a retrospective study. Data collected from medical and hospital records are not always complete and often underestimate complications and eventually overestimate the results. No specific urinary continence or sexual potency questionnaires were applied, which could provide more reliable data. Nevertheless, since we considered continent all patients who did not need any type of pad protection, we believe that the results were not significantly influenced by the absence of a specific questionnaire. Accordingly, we defined as potent those patients who had sexual intercourses with or without the aid of phosphodiesterase-5 blocking drugs. We believe that this also reduced the impact of the absence of a specific questionnaire regarding sexual potency. This is a pioneer study, as it comes from a private hospital in Brazil with no residence or fellow programs, analyzing a fairly recent technique that is not accessible to the majority of the patient and medical population. To our knowledge, this is also the first study reporting predictors of functional outcome recovery after RARP with more than one hundred patients.

## CONCLUSIONS

Age and obesity influenced the recovery of sexual potency, while only age was related to the recovery of urinary continence. We obtained good functional results, within the range of the largest published series, despite still within the learning curve. Prospective national studies with a larger number of patients are needed to better analyze functional results in larger Brazilian series.
